# The value of platelet-rich plasma in women with previous implantation failure: a systematic review and meta-analysis

**DOI:** 10.1007/s10815-023-02781-4

**Published:** 2023-04-03

**Authors:** Ahmed M. Maged, Akmal El-Mazny, Nada Kamal, Safaa I. Mahmoud, Mona Fouad, Noura El-Nassery, Amal Kotb, Wael S. Ragab, Asmaa I. Ogila, Ahmed A. Metwally, Radwa M. Fahmy, Hany Saad, Eman K. Shaeer, Noha Salah, Yossra Lasheen

**Affiliations:** 1 Department of Obstetrics and Gynecology, Kasr Al-Ainy Hospital, Cairo University, Cairo, Egypt; 2 Department of Obstetrics and Gynecology, Beni-Suef University, Beni-Suef, Egypt; 3 Department of Obstetrics and Gynecology, Fayoum University, Fayoum, Egypt

**Keywords:** Platelet-rich plasma, PRP, Autologous platelet-rich plasma, Implantation failure, Thin endometrium

## Abstract

**Objective:**

To assess the value of intrauterine PRP to improve IVF outcome in women with previous implantation failure.

**Methods:**

Screening of Pubmed, Web of Science, and other databases from inception to August 2022 using the keywords related to “platelet-rich plasma” OR “PRP” AND “IVF” “implantation failure.” Twenty-nine studies (3308 participants) were included in our analysis, 13 were RCTs, 6 were prospective cohorts, 4 were prospective single arm, and 6 were retrospective analyses. Extracted data included settings of the study, study type, sample size, participants’ characteristics, route, volume, timing of PRP administration, and outcome parameters.

**Results:**

Implantation rate was reported in 6 RCTs (886 participants) and 4 non-RCTs (732 participants). The odds ratio (OR) effect estimate was 2.62 and 2.06, with 95% CI of 1.83, 3.76, and 1.03–4.11, respectively. Endometrial thickness was compared in 4 RCTs (307 participants) and 9 non-RCTs (675 participants), which showed a mean difference of 0.93 and 1.16, with 0.59–1.27 and 0.68–1.65 95% CI, respectively.

**Conclusion:**

PRP administration improves implantation, clinical pregnancy, chemical pregnancy, ongoing pregnancy, live birth rates, and endometrial thickness in women with previous implantation failure.

**Supplementary Information:**

The online version contains supplementary material available at 10.1007/s10815-023-02781-4.

## Introduction

IVF failure is mostly related to implantation failure. Successful implantation requires a precisely synchronized development of both endometrium and blastocyst [1]. Optimized endometrial development requires cellular, vascular, and immunological modifications [2].

These changes include the replacement of the endometrial stromal cells by the decidual cells. The latter is characterized by the development of apical projections (pinopodes), glandular growth, and the development of microvilli on the endometrial luminal epithelial surface [3]. These cellular changes are associated with modifications of adhesion molecules, cytokines, growth factors, and loss of inhibitory mediators, resulting in vascular invasion and endometrial immune cell infiltration [4].

Various interventions have been tried to improve implantation, especially for those with repeated implantation failure (RIF). These interventions include endometrial scratch injury [5], hysteroscopic correction of cavity pathology [6], improving endometrial thickness in women with thin endometrium [7, 8], intrauterine administration of autologous peripheral blood mononuclear cells [9], human chorionic gonadotropin [10], granulocyte colony-stimulating factor [11], growth hormone [12], intravenous Atosiban [13], and the use of immunomodulators [14]. However, even with these new treatment approaches, many patients still suffer from RIF. Therefore, there is a need for an alternative treatment with more success in patients with a history of treatment failure.

Platelet-rich plasma (PRP), also known as autologous conditioned plasma, is a concentrate of platelet-rich blood prepared through centrifugation of fresh whole blood to remove red and white blood cells. The resultant precipitate is rich in growth factors and cytokines (e.g., VEGF, TGFβ, and PDGF) released from activated platelets α-granules [15]. PRP has regenerative and anti-inflammatory characteristics and has been used in various medical fields, such as ophthalmology and orthopedics [16].

It was first applied to improve refractory endometrium by Chang in 2015 [17]. Since then, it has been studied in the treatment of female infertility in women with RIF, thin endometrium, premature ovarian failure, and Asherman syndrome. The results of these studies revealed conflicting findings, especially in those with implantation failure and thin endometrium [9]. This systematic review and meta-analysis aimed to assess the value of intrauterine PRP to improve IVF outcomes in women with previous implantation failure.

## Material and methods

A prospectively prepared protocol that follows the Preferred Reporting Items for Systematic Reviews and Meta-Analyses (PRISMA) guidelines for meta-analysis was registered at PROSPERO. The registration number was CRD42022327811.

### Eligibility criteria, information sources, and search strategy

Two authors (AM, AE) searched Pubmed, Web of Science, Scopus, and the Cochrane Central Register of Controlled Trials electronic databases from inception to August 2022 using the keywords “platelet-rich plasma” OR “PRP” OR “autologous platelet-rich plasma”) AND “IVF” “ICSI” “implantation failure” OR “thin endometrium” and their MeSH terms. Abstracts of conferences, Google Scholar, and reference and citation lists of the subject-related studies were checked for any additional studies. Contacting the authors was done if any clarifications or additional data were needed through emails. Details about the search strategy are provided in Supplementary Table 1.

### Study selection

All available studies—with no language limitations—involved PRP administration around the time of embryo transfer in IVF/ICSI cycles. The types of studies included randomized controlled trials (RCTs) mainly. A separate analysis for cohort or single-arm studies, whether prospective or retrospective, was done. Studies compared PRP to no intervention, placebo, or granulocyte colony-stimulating factor (GCSF) were included. All routes of administration, whether intrauterine or subendometrial, were also included. Excluded studies included in vitro (cell culture) studies, animal studies, case reports, and studies with an inadequate methodology or unclear outcomes (and cannot be clarified by author correspondence).

### Data extraction

Two authors (AM and NS) examined the search results titles and abstracts according to the predetermined eligibility criteria and then evaluated the full articles of the related studies. Any disagreement between the 2 authors regarding inclusion was discussed with other co-authors. Data from selected articles were extracted independently by 2 authors (AM and NS), and disagreement was dealt with in the same way as inclusion. Extracted data included settings of the study, sample size, participants’ inclusion and exclusion criteria, intervention characteristics, outcome parameters, registration, and funding data. The authors were contacted to clarify any vague or missed data.

### Assessment of risk of bias

Quality assessment of the included RCTs was done following the Cochrane Handbook of Systematic Reviews recommendations by two investigators (AM and MF), and disagreements were discussed further with other investigators. All studies were assessed for random sequence generation, allocation concealment, blinding of participants and outcome assessors, incomplete outcome data, selective reporting, and other bias were done.

Quality assessment of non-RCTs was done using the Newcastle–Ottawa scale (NOS). This “star system” is based on three main perspectives: the selection of the study groups (exposed and non-exposed); the comparability of the groups (cohorts or cases and controls) on the basis of the design or analysis; the ascertainment of exposure or outcome (length and adequacy of follow-up). Absent and unclear data were checked by contacting the corresponding author or other coauthors.

The GRADE system was used to assess the quality of evidence. GRADE included the risk of bias in the included studies due to inconsistency, indirectness, imprecision, and publication bias. Serious concerns in each item decrease the evidence by 1 level, while very serious ones decrease the evidence by 2 levels.

The levels were high, moderate, low, or very low if we were very confident, moderately confident, have limited confidence, or very limited confidence that the true effect is close to the effect estimate, respectively.

### Data synthesis

The odd ratio and the corresponding 95% CI were calculated for all dichotomous data, and the mean difference with the corresponding 95% CI was calculated for continuous data. The effect size was obtained using the random effect model by the Mantel–Hansel method.

The heterogeneity of studies included was evaluated by I2 statistic and Cochran’s Q test. Heterogeneity was considered significant at a *p*-value of < 0.05 in the Q-test or I2 > 40%. A separate analysis was done for RCTs and non-RCTs, and subgroup analysis of the studies according to inclusion criteria of participants (previous implantation failure or thin endometrium), the volume of transferred PRP (ranged from 0.5 to 40 ml), and types of transferred embryos (fresh or frozen). All statistical analysis was performed with the Review Manager (RevMan) version 5.4.1 (The Nordic Cochrane Centre, Cochrane Collaboration 2020; Copenhagen, Denmark).

## Results

### Study selection

**Fig. 1:**
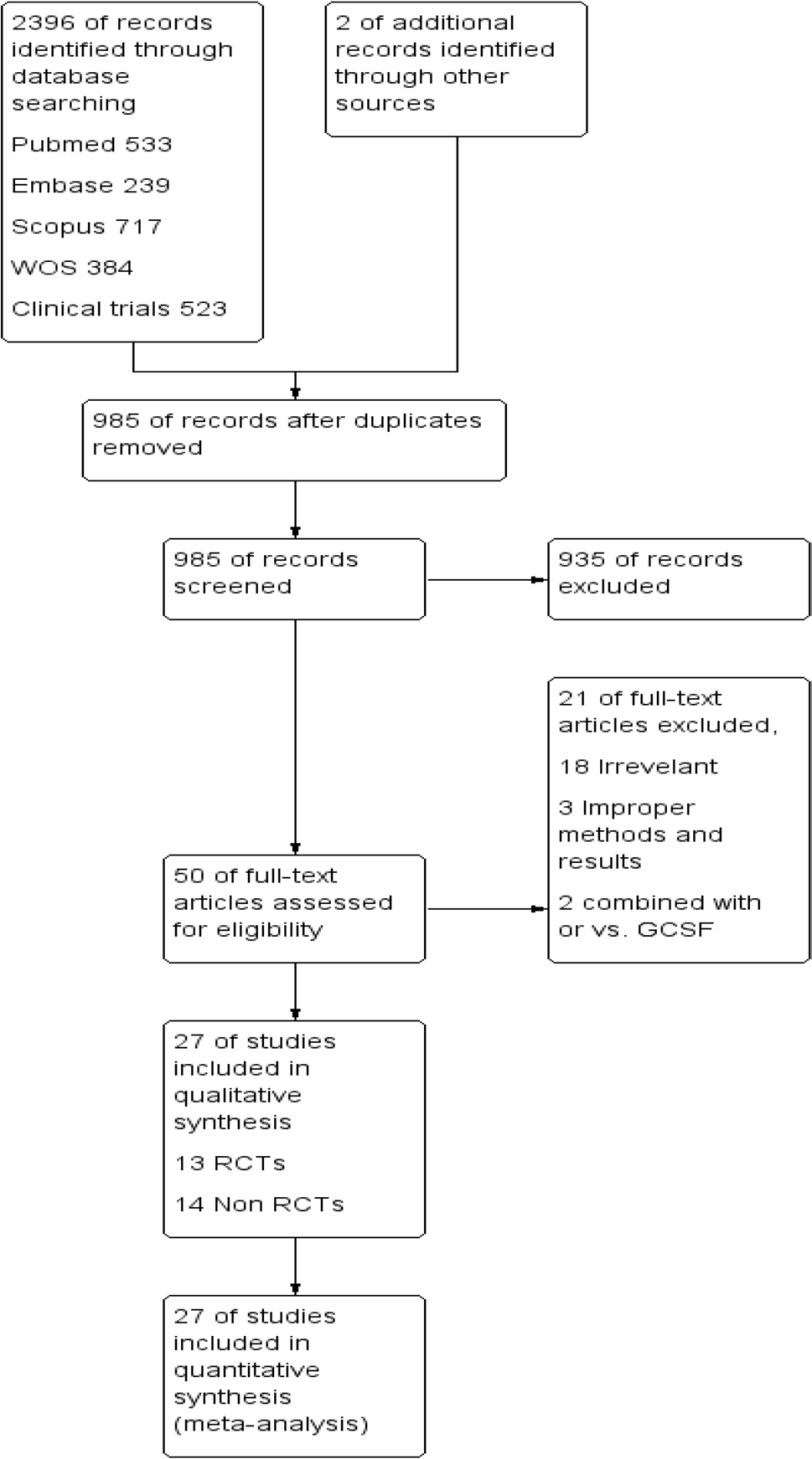
PRISMA flow chart

Our search yielded 2398 studies through databases (533 from PubMed, 239 from Embase, 717 from Scopus, 384 from Web of Science, 523 from clinical trials, and 2 through other sources), 985 of them were screened after the removal of duplicates, 50 screened for full text, and 29 studies were included in quantitative and qualitative synthesis (Fig. 1).

### Study characteristics

Tables S2 and S3 summarized the main characteristics of the included RCTs and non-RCTs. Twenty-seven studies were included in our analysis: 13 were RCTs [18–30], 5 was prospective cohort [17, 31–35], 4 were prospective single-arm [35–38], and 5 were retrospective analysis [39–44]. All the studies were single centers except Kusumi [35], which was conducted in 7 fertility clinics. Eleven studies were conducted in Iran, 3 in Russia [22, 29, 34], 3 in China [17, 36, 39], 2 in India [38, 45], 2 in Japan [35, 41], and 1 study was conducted in each of the following countries: Bahrain [28], Canada [40], Egypt [20], South Korea [37], Turkey [42], and UK [32]. In 12 studies, the participants had recurrent implantation failure, in 5 studies had implantation failure, and in 10 studies, had thin endometrium. Frozen embryo transfer was done in 22 studies, fresh embryo transfer cycles were investigated in 2 studies [28, 34], while in 3 studies the cycles included had both fresh and frozen embryo transfer [20, 27, 38]. All the studies evaluated intrauterine injection except Apolikhina [21], which evaluated subendometrial injection and Nourshin [32], which evaluated both intrauterine and subendometrial PRP injections. PRP was compared to no intervention or placebo in all 27 studies. PRP volume injected was 0.3–0.4 ml in 1 study [45], 0.5–1 ml in 22 studies, 1.5 ml in 1 study [27], 2 ml in 1 study [22], 5–7 ml in 1 study [34], and 35–40 ml in 1 study [29]. The timing of PRP injection was 48 h before ET in 12 studies, between cycle days 6 and 14 in 12 studies, unspecified time in 2 studies, and when the endometrial thickness was below 7 mm in 1 study.

PRP preparation in the included studies was achieved through a two-step process. Venous blood was added to acid citrate and centrifugated for about 10 min to separate the red and white blood cells, then centrifugated again to reach 4–5 times platelet concentration.

### Risk of bias of included studies

**Fig. 2:**
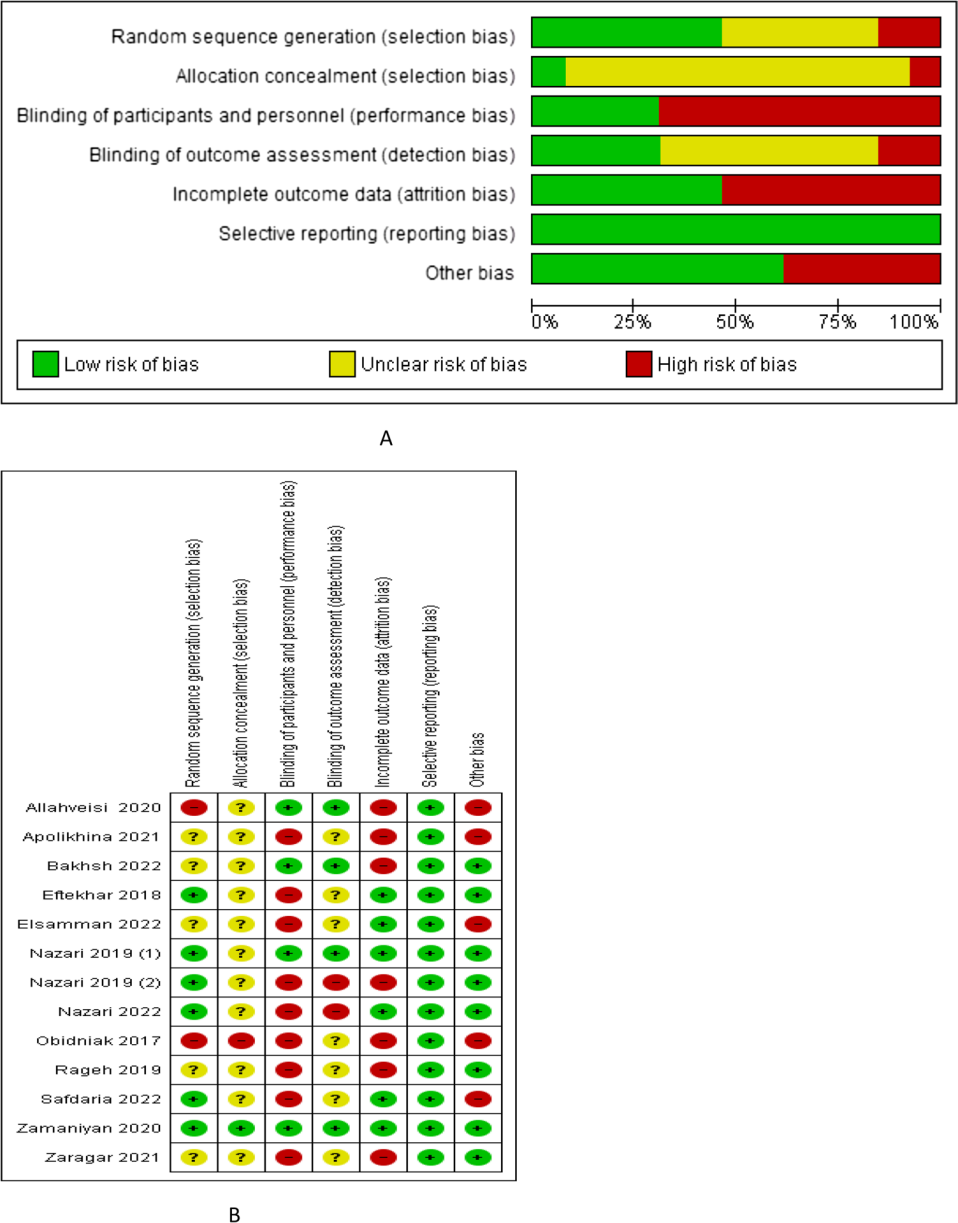
Risk of bias A graph and B summary

Quality assessment of the included RCTs was done following the Cochrane Handbook of Systematic Reviews; recommendation is shown in Fig. 2. Quality assessment of the included non-RCTs was done using Newcastle–Ottawa Scale, is summarized in Table 1. GRADE quality of evidence for each outcome criteria is summarized in Table 2.

**Table 1:** Quality assessment of the included non RCTs using Newcastle-Ottawa Scale

[Study]	Selection	Comparability	Outcome/exposure
Noushin 2021	***	*	***
Chang 2019	***	*	***
Dzhincharadze 2021	***	*	***
Tehraninejad 2020	***	*	***
Kim 2019	***	---	***
Kusumi 2020	***	---	***
Dogra 2022	***	---	***
Wang 2018	**	---	*
Zadehmodarres 2017	**	----	*
Madhavan 2018	***	*	**
Xu 2022	***	*	**
Coksuer 2019	***		***
Enatsu 2021	***	*	**
Russell 2022	***	*	**

**Table 2:** GRADE quality of evidence

Outcome	No studies	Risk of bias	Inconsistency	Indirectness	Imprecision		Publication bias	Quality
					Sample size	Wide CI		
Implantation rate	6	N	S	N	886	N	N	Moderate
Clinical pregnancy rate	11	S	S	N	1289	N	N	Low
Chemical pregnancy rate	7	N	N	N	726	N	N	High
Ongoing pregnancy rate	5	N	S	N	488	N	N	Low
Live birth rate	4	S	S	N	523	S	N	Very low
Endometrial thickness	4	S	N	N	307	N	N	Low

*CI*, confidence interval; *N*, not serious; *S*, serious

### Synthesis of results

**Fig. 3:**
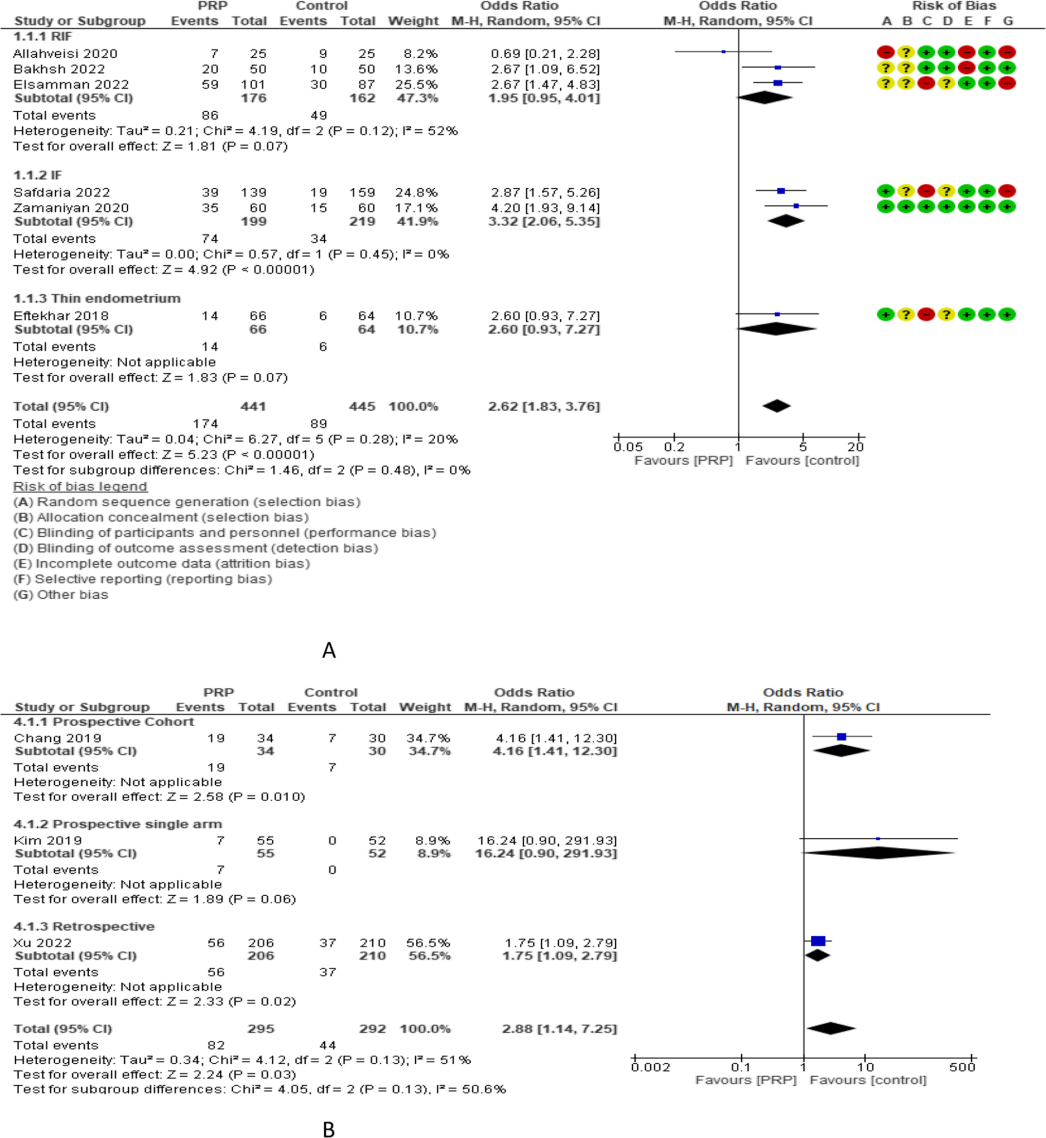
Implantation rate in A RCTs and B non-RCTs

Implantation rate (IR) was reported in 6 RCTs with 886 participants. The odd ratio effect estimate was 2.62 with a 95% CI of [1.83, 3.76]. Subgroup analysis reported IR in 3 studies (338 women) with repeated implantation failure and revealed an overall estimated OR of 1.95 and 95% CI of 0.95–4.01. IR was reported in 2 and 1 studies (418 and 130 women) with previous implantation failure and thin endometrium and revealed overall estimated OR of 3.32 and 2.60 with 95% CI of 2.06–5.35 and 0.93–7.27, respectively. IR was reported in 5 and 1 studies (698 and 188 women) with frozen and both frozen and fresh embryo transfer and revealed overall estimated OR of 2.54 and 2.67 with 95% CI of 1.57–4.11 and 1.47–4.83, respectively. IR was evaluated in 3 non-RCTs with 587 participants and revealed an overall estimated OR of 2.88 with a 95% CI of 1.14–7.25 [1 study (64 women) was prospective cohort and had 4.16 OR and 1.41–12.30 95% CI, 1 study (107 women) was prospective single arm and had 16.24 OR and 0.9–291.93 95% CI, and 1 study (416 women) was retrospective and had 1.75 OR and 1.09–2.79 95% CI (Fig. 3).

**Fig. 4:**
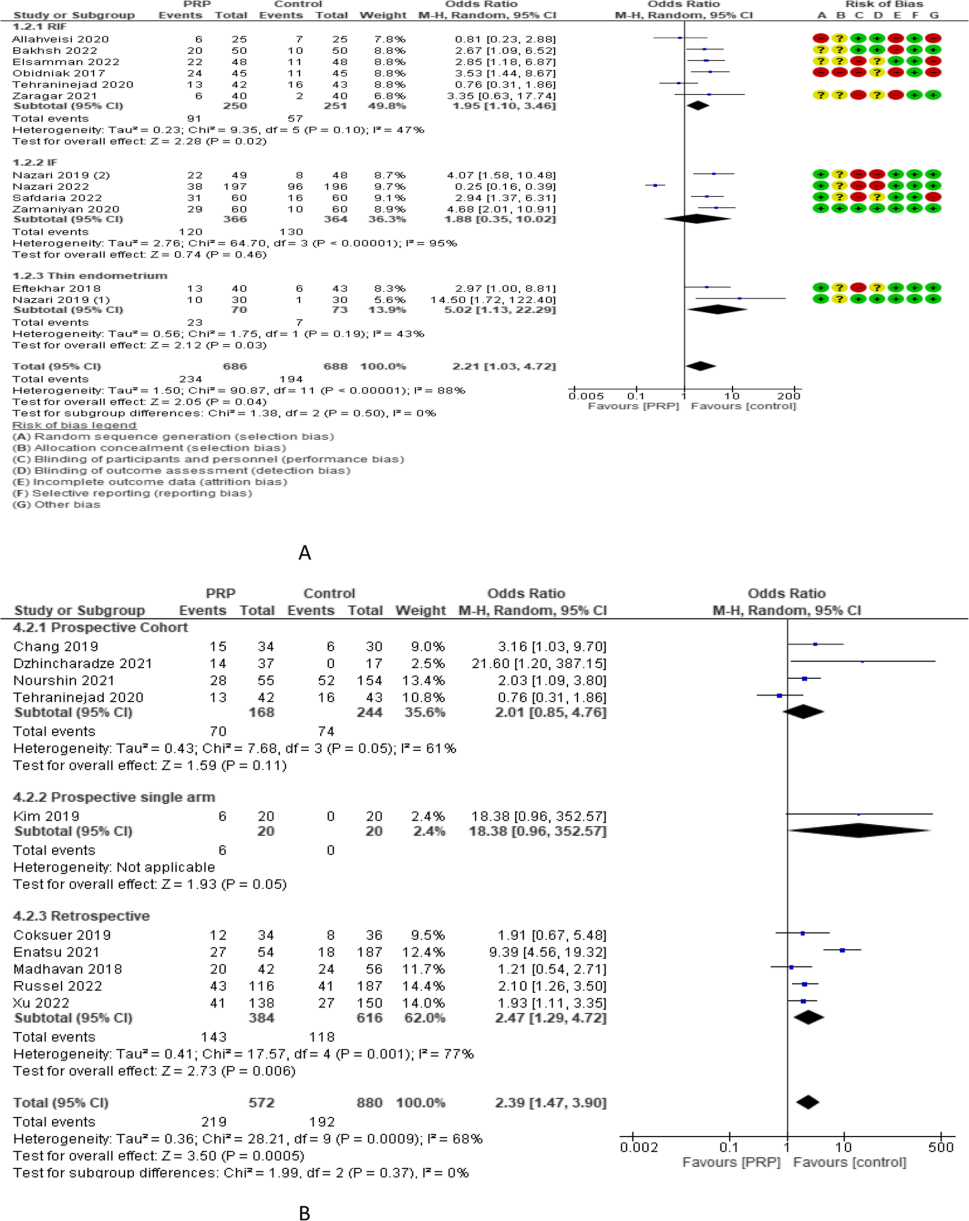
Clinical pregnancy rate in A RCTs and B non-RCTs

Clinical pregnancy rate (CPR) was reported in 11 RCTs with 1289 participants. The odd ratio effect estimate was 2.46 with a 95% CI of [1.08, 5.63]. Subgroup analysis reported CPR in 5 studies (416 women) with repeated implantation failure and revealed an overall estimated OR of 2.54 and 95% CI of 1.61–4.02. CPR was reported in 4 and 2 studies (730 and 143 women) with previous implantation failure and thin endometrium and revealed overall estimated OR of 1.88 and 5.02 with 95% CI of 0.35–10.02 and 1.13–22.29, respectively. CPR was reported in 9 and 2 studies (1113 and 176 women) with frozen and both frozen and fresh embryo transfer and revealed overall estimated OR of 2.37 and 2.95 with 95% CI of 0.91–6.20 and 1.35–6.43, respectively. Subgroup analysis of CPR according to the volume of PRP injected revealed that 0.5–1 ml (9 studies, 1119 women), 1.5 ml (1 study, 80 women), and 2 ml (1 study, 90 women) had a CPR OR of 2.31, 3.35, and 3.53 and 95% CI of 0.89–5.96, 0.63–17.74, and 1.44–8.67, respectively. CPR was evaluated in 10 non-RCTs with 1452 participants and revealed an overall estimated OR of 2.39 with a 95% CI of 1.47–3.90 [4 studies (412 women) were prospective cohort and have 2.01 OR and 0.85–4.76 95% CI, 1 study (40 women) was prospective single arm and have 18.38 OR and 0.96–352.57 95% CI, and 5 studies (1000 women) were retrospective and have 2.47 OR and 1.29–4.72 95% CI (Fig. 4).

**Fig. 5:**
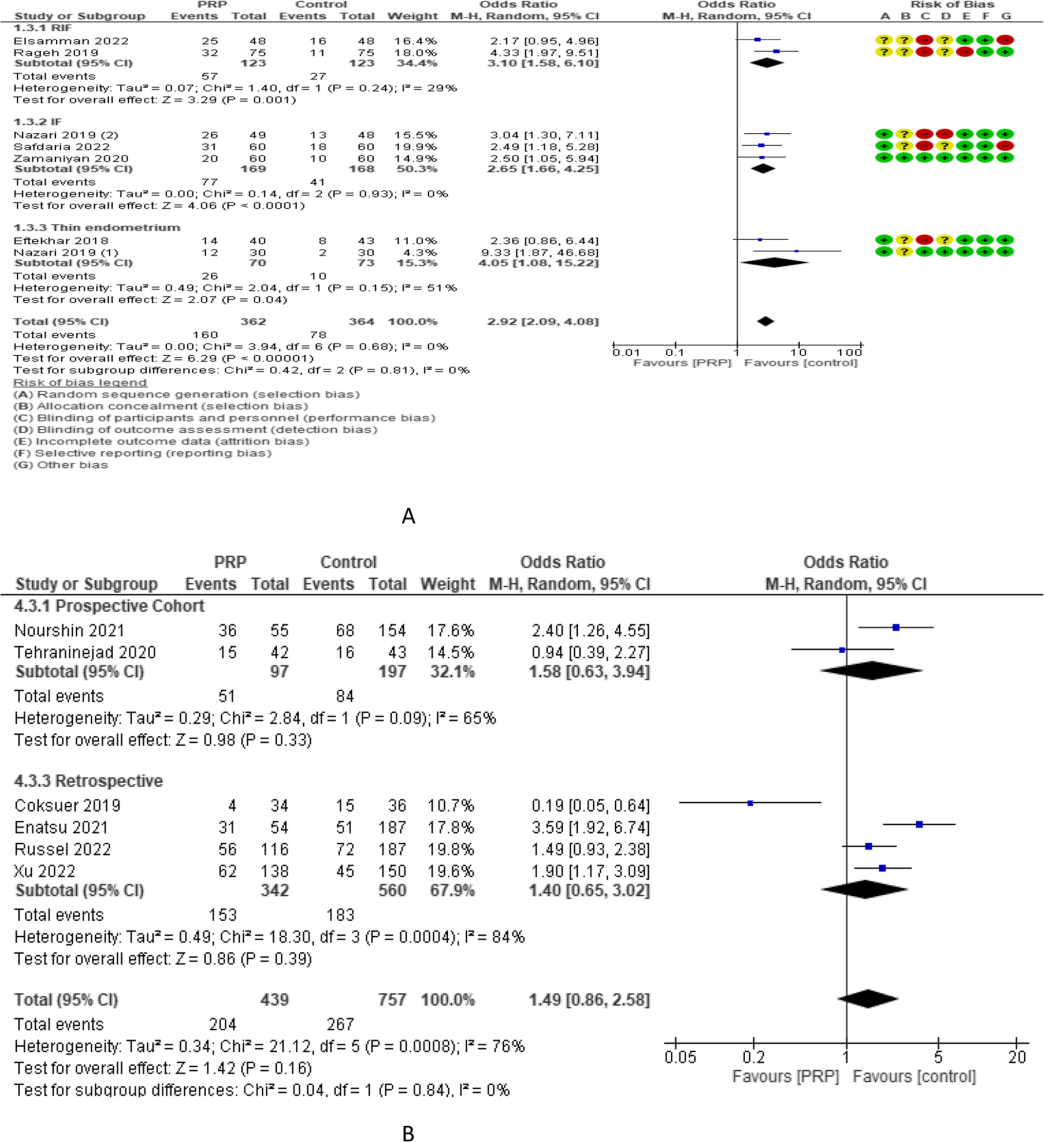
Chemical pregnancy rate in A RCTs and B non-RCTs

Chemical pregnancy rate was reported in 7 RCTs with 726 participants. The odd ratio effect estimate was 2.92 with a 95% CI of [2.09, 4.08]. Subgroup analysis reported chemical pregnancy rate in 2 studies (246 women) with repeated implantation failure and revealed an overall estimated OR of 3.10 and 95% CI of 1.58–6.10. Chemical pregnancy rate was reported in 3 and 2 studies (337 and 143 women) with previous implantation failure and thin endometrium and revealed overall estimated OR of 2.65 and 4.05 with 95% CI of 1.66–4.25 and 1.08–15.22, respectively. Chemical pregnancy rate was reported in 5.1 and 1 studies (480,150 and 96 women) with frozen, fresh, and both frozen and fresh embryo transfer and revealed overall estimated OR of 2.83, 4.33, and 2.17 with 95% CI of 1.87–4.27, 1.97–9.51, and 0.95–4.96 respectively. The chemical pregnancy rate was evaluated in 6 non-RCTs with 1196 participants and revealed an overall estimated OR of 1.49 with a 95% CI of 0.86–2.58 [2 studies (294 women) was prospective cohort and has 1.58 OR and 0.63–3.94 95% CI and 4 studies (902 women) were retrospective and has 1.40 OR and 0.65–3.02 95% CI (Fig. 5).

**Fig. 6:**
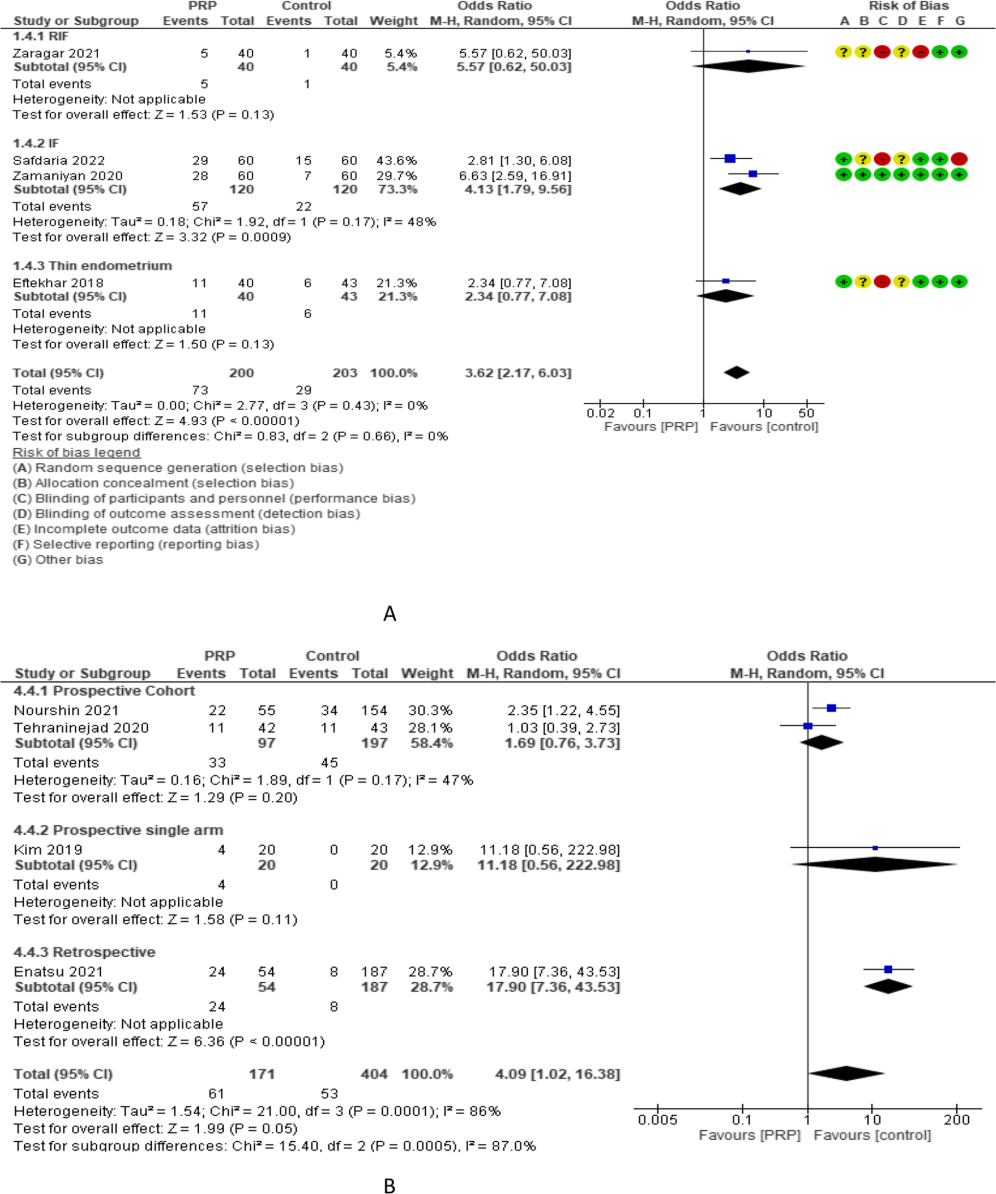
Ongoing pregnancy rate in A RCTs and B non-RCTs

Ongoing pregnancy rate (OPR) was reported in 5 RCTs with 488 participants. The odd ratio effect estimate was 2.78, with a 95% CI of [1.43, 5.41]. Subgroup analysis reported OPR in 2 studies (165 women) with repeated implantation failure and revealed an overall estimated OR of 1.79 and 95% CI of 0.37–8.53. OPR was reported in 2 and 1 studies (240 and 83 women) with previous implantation failure and thin endometrium and revealed overall estimated OR of 4.13 and 2.34 with 95% CI of 1.79–9.56 and 0.77–7.08, respectively. OPR was reported in 4 and 1 studies (408 and 80 women) with frozen and both frozen and fresh embryo transfer and revealed overall estimated OR of 2.62 and 5.57 with 95% CI of 1.26–5.47 and 0.62–50.03, respectively. Subgroup analysis of OPR according to the volume of PRP injected revealed that 0.5–1 ml (4 studies, 408 women) and 1.5 ml (1 study, 80 women) had an OPR OR of 2.62 and 5.57 and 95% CI of 1.26–5.47 and 0.62–50.03, respectively. OPR was evaluated in 4 non-RCTs with 575 participants and revealed an overall estimated OR of 4.09 with a 95% CI of 1.02–16.38 [2 studies (294 women) were prospective cohort and had 1.69 OR and 0.76–3.73 95% CI, 1 study (40 women) was prospective single arm and had 11.18 OR and 0.56–222.98 95% CI, and 1 study (241 women) was retrospective and had 17.90 OR and 7.36–43.53 95% CI (Fig. 6).

**Fig. 7:**
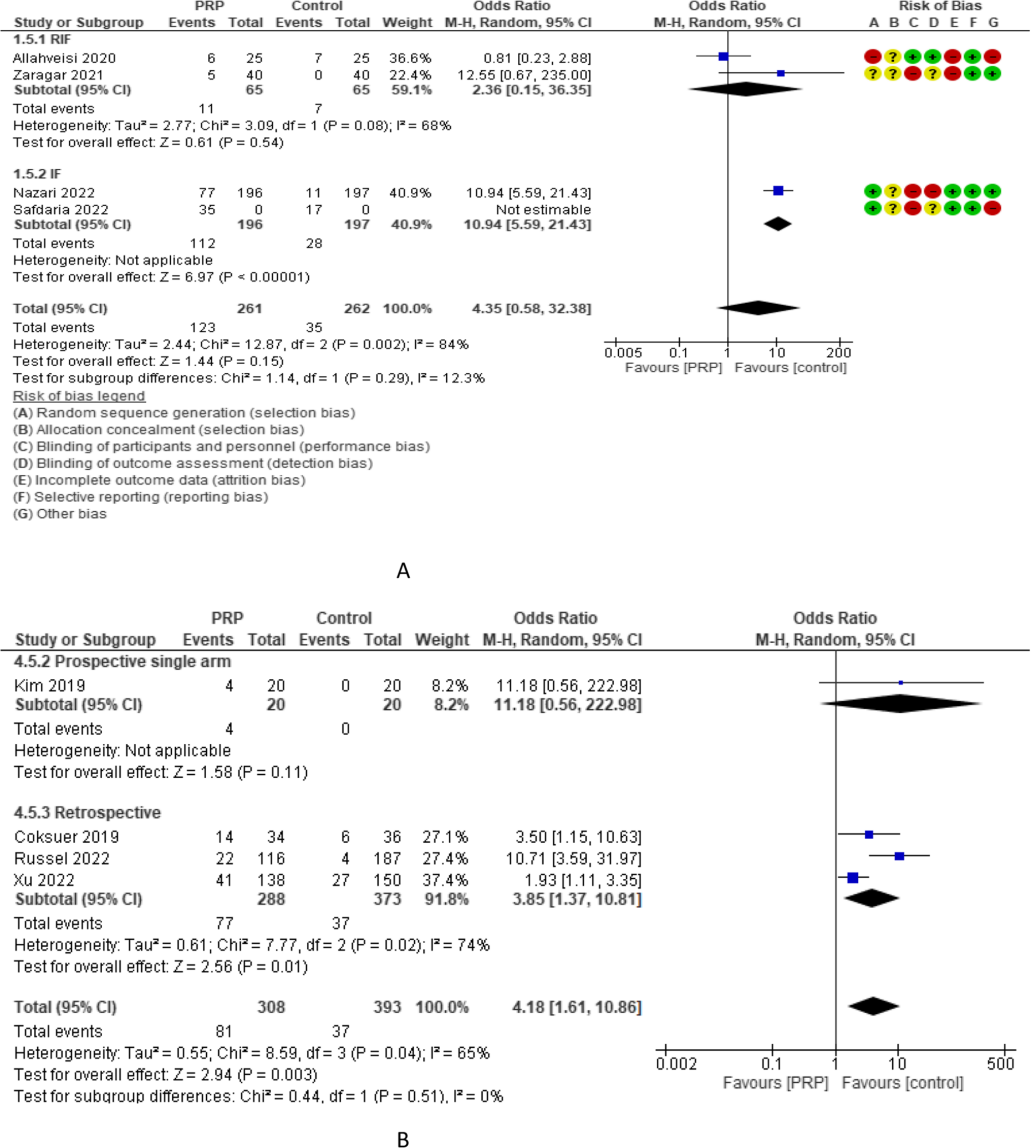
Livebirth rate in A RCTs and B non-RCTs

Live birth rate (LBR) was reported in 4 RCTs with 523 participants. The odd ratio effect estimate was 4.35 with a 95% CI of [0.58, 32.38]. LBR was reported in 2 studies (130 women) with repeated implantation failure and 2 studies (393 women) with previous implantation failure and revealed overall estimated OR of 2.36 and 10.94 with 95% CI of 0.15–36.35 and 5.59–21.43, respectively. LBR was reported in 3 studies (563 women) with frozen embryo transfer and 1 study (80 women) with both frozen and fresh embryo transfer and revealed overall estimated OR of 3.47 and 12.55 with 95% CI of 0.93–12.91 and 0.67–235.00, respectively. Subgroup analysis of LBR according to the volume of PRP injected revealed that 0.5–1 ml (3 studies, 563 women) and 1.5 ml (1 study, 80 women) had an LBR OR of 3.47 and 12.55, and 95% CI of 0.93–12.91 and 0.67–235.00, respectively. LBR was evaluated in 4 non-RCTs with 701 participants and revealed an overall estimated OR of 4.18 with a 95% CI of 1.61–10.86 [1 study (40 women) was prospective single arm and had 11.18 OR and 0.56–222.98 95% CI and 3 studies (661 women) were retrospective and had 3.85 OR and 1.37–10.81 95% CI (Fig. 7).

**Fig. 8:**
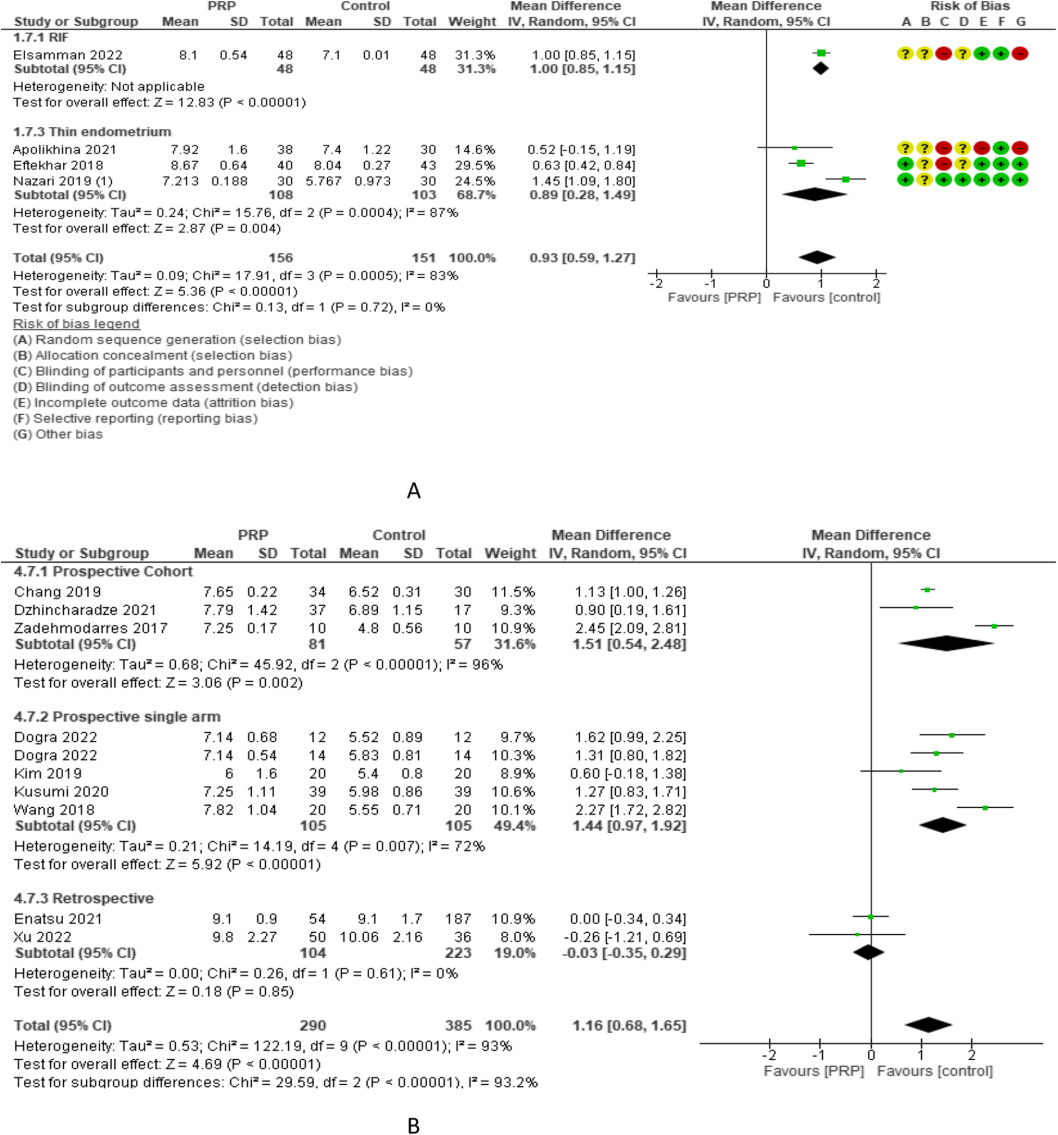
Endometrial thickness in A RCTs and B non-RCTs

Endometrial thickness was compared in 4 RCTs with 307 participants and showed a mean difference of 0.93 with 0.59–1.27 95% CI between PRP and control cycles. Endometrial thickness was reported in 1 [23] and 3 studies [21, 43, 46] (96 and 211 women) with repeated implantation failure and thin endometrium and revealed an overall estimated mean difference of 1.00 and 0.89 with 95% CI of 0.85–1.15 and 0.28–1.49, respectively. It was also reported in 3 and 1 studies (211 and 96 women) with frozen and both frozen and fresh embryo transfer and revealed an overall estimated mean difference of 0.89 and 1.00 with 95% CI of 0.28–1.49 and 0.85–1.15, respectively. Subgroup analysis of LBR according to the volume of PRP injected revealed that 0.5–1 ml (3 studies, 239 women) and 35–40 ml (1 study, 68 women) had an endometrial thickness mean difference of 1.00 and 0.52 and 95% CI of 0.63–1.38 and − 0.15 to 1.19, respectively. Endometrial thickness was evaluated in 9 non-RCTs with 675 participants and revealed an overall estimated mean difference of 1.16 mm with a 95% CI of 0.68–1.65 mm [3 studies (138 women) in were prospective cohort and had 1.51 mm mean difference and 0.54–2.48 mm 95% CI, 4 studies (210 women) were prospective single-arm and had 1.44 mm mean difference and 0.97–1.92 mm 95% CI, and 2 studies (327 women) were retrospective and had − 0.03 mean difference and − 0.35 to 0.29 mm 95% CI (Fig. 8).

Two prospective cohort studies by Apolikhina et al. [21] and Noushin et al. [32] evaluated the effects of subendometrial injection of PRP. Apolikhina’s study involved 68 women with a history of cycle cancellation resulting from refractory thin endometrium not responding to the standard treatment. Thirty-eight women were treated by physical electropulse therapy with abdominal and vaginal placement of electrodes on “BTL-4000 Premium G’” unit from cycle days 5–7 for 10 days and then received subendometrial injection of 35–40 ml of autologous PRP during the cycle next to physical therapy. The injection was done by endoscopic needle through hysteroscopy. They were compared to 30 women who were treated only by physical therapy. Endometrial growth was significantly higher in the PRP group compared to controls (7.92 ± 1.6 vs 7.4 ± 1.22, *p*-value < 0.001, obtained by author contact). They concluded that PRP injection is effective in women with refractory “thin” endometrium and decreased uterine artery hemodynamics.

Noushin et al. study [32] included women with recurrent implantation failure undergoing frozen embryo transfer. They compared 55 women subjected to ultrasonographic guided transvaginal subendometrial injection of PRP during the luteal phase of the cycle prior to the embryo transfer cycle under ultrasound guidance and 109 women subjected to intrauterine PRP injection done during the embryo transfer cycle at an approximate endometrial thickness of 7 mm to 154 women who underwent standard cycle without intervention. Women in the 2 intervention groups received additional subcutaneous injections of 300 mg of GCSF daily for 3 days. They found that ongoing/livebirth rates were higher in the intervention groups compared to the control group [22/55 (40%), 45/109 (41.3%), and 34/154 (22.1%), respectively; *p* = 0.004]. They reported a similarly higher clinical pregnancy rate [28/55 (51%), 57/109 (52.3%) vs 52/154 (33.8%), respectively; *p* = 0.006]. They concluded that PRP improves the outcome of frozen embryo transfer cycles with no difference between subendometrial injection and intrauterine infusion.

## Discussion

### Main findings

This meta-analysis found a beneficial effect of PRP administration on implantation, clinical pregnancy, chemical pregnancy, ongoing live birth rates, and endometrial thickness. These effects on IVF outcomes were constant with changing types of participants, types of embryos transferred, and volume of PRP injected in both RCTs and non-RCTs. However, the quality of evidence of these findings was very low regarding live birth rate (only 4 studies with 523 participants), low in endometrial thickness (4 studies with 307 participants), and clinical pregnancy rate (high risk of bias with the inconsistency of results), moderate in implantation rate and high in chemical pregnancy rate.

The possible mechanisms of the beneficial effects of PRP include synchronization of immunological interactions between the endometrial and embryo development during the implantation window. PRP decreases inflammatory cytokines such as IL-6 and 8 and increases IL-1 β, which is crucial in successful implantation [44].

However, the exact mechanism is still not clear, and these beneficial effects may result from mechanical endometrial injury caused by intrauterine catheter simulating endometrial injury effects, as in most of the included studies, the control group had no intervention.

It is to be noted that the CI is passing through 1 in LBR, endometrial thickness, and chemical pregnancy rate.

### Strengths and limitations

Our meta-analysis is the first comprehensive one focusing on the effects of PRP in women with previous implantation failure. It included all the available studies reached by extensive searching of all available databases and the gray literature, trial registration sites, and a reference list of all related studies. A separate analysis for RCTs and non-RCTs was done. Adequate subgroup analysis according to participants’ characteristics, route, and volume of PRP injected for all the available outcomes.

This meta-analysis is not without limitations. Only 11 RCTs were included. Most of them had a high risk of bias, especially in blinding and allocation concealment. The most important outcome (live birth rate) was reported in 4 studies only. Most of the included studies did not describe accurate details about PRP preparation. The exact cause of previous implantation failure was not clarified in most studies except those that described thin endometrium. The studies describing thin endometrium failed to describe the exact method of measuring the endometrial thickness and the presence of any intra- or inter-observer variability. The exact timing and number of PRP administrations were not clear in most of the studies. These led to marked heterogeneity. We tried to compensate for that by using the random effect method for comparison.

### Comparison with existing literature

There are many systematic reviews evaluating the effects of PRP in orthopedic, ophthalmic, and dermatological fields. Few ones were done in gynecology. Some evaluated its role in Asherman syndrome and others evaluated it in premature ovarian failure. Only 1 systematic review studied its role in implantation.

Maleki-Hajiagha and colleagues [9] evaluated the effect of PRP on the outcome of embryo transfer in IVF/ICSI. They included 7 studies with 625 women (311 cases vs. 314 controls). They reported higher clinical pregnancy (7 studies, RR: 1.79, 95% CI: 1.37, 2.32; *P* < 0.001, I2 = 16%), chemical pregnancy (3 studies, RR 1.79, 95% CI: 1.29, 2.50; *P* < 0.001, I2 = 0%), and implantation rates (*n* = 3, RR: 1.97, 95% CI: 1.40, 2.79; *P* < 0.001, I2 = 0%). They also reported more increase in endometrial thickness in women who received PRP compared to the control group (SMD: 1.79, 95% CI: 1.13, 2.44; *P* < 0.001, I2 = 64%). They concluded that PRP could be used as an accessory strategy in women with RIF and thin endometrium. However, this review included only 3 RCTs and 4 cohort studies. So, the subgroup analysis was defective.

## Conclusions

This systematic review showed an increase in all outcomes of IVF cycles, namely implantation, clinical pregnancy, chemical pregnancy, ongoing pregnancy, and live birth rates. It also reported a significant increase in endometrial thickness in women with refractory thin endometrium. However, the quality of evidence was generally low, as the number of well-designed RCTs was inadequate to provide strong evidence, and there was marked heterogeneity among the included studies. More RCTs with adequate blinding, low risk of bias, with precise inclusion criteria considering the possible causes of implantation failure and other markers of endometrial receptivity besides the endometrial thickness should be conducted to provide the needed evidence.

## Supplementary information

ESM 1: Supplementary Tables
