# Supplementary material for: The value of platelet-rich plasma in women with previous implantation failure: a systematic review and meta-analysis
Source: J Assist Reprod Genet. 2023 Apr 3;40(5):969–83. doi: 10.1007/s10815-023-02781-4 (PMC10239431; doi:10.1007/s10815-023-02781-4)
Supplement: Supplementary file 1 — Supplementary Tables [file 10815_2023_2781_MOESM1_ESM.docx]

Supplementary tables

Table S1 search strategy

| Data base | keywords | Number of studies |
| --- | --- | --- |
| Pubmed | Title, Abstract, Keywords search ( in vitro fertilization OR IVF OR ICSI OR Intracytoplasmic sperm injection OR Assisted reproductive techniques OR ART OR Implantation failure OR repeated implantation failure OR Recurrent implantation failure OR failed implantation) AND ( platelet-rich plasma OR platelet rich plasma OR PRP OR autologous platelet-rich plasma OR platelet rich plasma gel OR PRP gel) | 533 |
| EMBASE |  | 239 |
| Scopus |  | 717 |
| WOS |  | 384 |
| Clinical trials |  | 523 |

Table S2 characteristics RCT

| [Study] | [Settings] | [Sample size] Fresh or frozen | [Participants] RIF or no | PRP preparation | [Interventions] No & volume | [Outcomes] | [Notes] |
| --- | --- | --- | --- | --- | --- | --- | --- |
| Allahveisi 2020 | Single center Iran | 50 frozen | Inclusion criteria  History of failed implantation candidate for frozen ET  Exclusion criteria  Unclear | Volume: 35 mL of venous blood coated with 5 cc of acid citrate as the anticoagulant solution (ACD-A; Iran)  Centrifugation: 1700 G for 12 min in an aseptic PRP centrifuge kit (ROOYA GEN PRP; Iran). Thereafter, the separated plasma was centrifuged for 7 min at 3300 G  Platelet concentration: 411X10^3^ - 1067X10^3^/μL | Control group (25 women)  slow intrauterine infusion of 0.5 mL of Ringer serum was done 48 h before ET  Intervention group (25 women)  the slow intrauterine infusion of 0.5 mL of PRP was performed 48 h before ET | Implantation rate  Clinical pregnancy rate  Live birth rate | No registration  No funding |
| Bakhsh 2022 | Single center Iran | 100 frozen | Inclusion criteria  e infertile women with a history of RIF candidates for IVF/ ICSI or freeze embryo transfer cycles ; age < 40 years; and BMI < 30kg/m2, FSH >12 IU  Exclusion criteria  hematological and immunological disorders; cancers; hormonal disorders; Hb <11 g/dl, platelets <150,000mm3, chromosomal and genetic abnormalities, receiving drugs (anticoagulants, NSAID in last 7 days, predinisolone, IVIG, GCSF), smokers, uterine anomalies, uncontrolled diseases as DM or hypertension, simultaneous endometrial scratch | Protocol: Two-step centrifuging process  Volume: 8.5 mL of venous blood added to 2.5 cc of acid citrate as the anticoagulant solution (Royagen / Aria Mabna Tashkhis, Iran)  Centrifugation: centrifuged for 10 minutes at 1400 rpm to separate the red blood cells. The upper plasma was then separated and centrifuged again at 3500 rpm for another 6 minutes.  Platelet concentration: 4-5 times higher than its amount in the peripheral blood | Control (50 women)  the catheter was transferred and removed without injection or any other action  Intervention (50 women)  0.5 cc of PRP was injected into the uterine cavity through an IUI transfer catheter | Implantation rate  Clinical pregnancy rate | N22017073034422  No funding |
| Elsamman 2022 | ?? centers Egypt | 96 women | Inclusion criteria  Age 18-35 years  RIF ≥ 3 (poor endometrium)  Exclusion criteria  Platelets < 150000/mm3  receiving drugs (anticoagulants, NSAID in last 10 days)  major comorbidities or psychiatric illness which might threaten the patient 's consent. Active cervical or uterine infection, Women with recognized implantation failure reason, like poor embryo quality, Asherman syndrome, or congenital uterine abnormalities | Protocol:  Volume: 10 cm3 sample is withdrawn in a syringe with an anticoagulant  Centrifugation: sequential centrifugation (soft spin 200 g/15 min, then hard spin 600 g/6 min) | Control group (48 women) 24 frozen and 24 fresh ET  No intervention  Intervention group (48 women) 24 frozen and 24 fresh ET  A 48 h prior to ET, a 0.5 ml intrauterine infusion of PRP containing 4-5 times more platelets than a peripheral blood sample is given using an IUI cannula and ultrasound guidance | Implantation rate  Clhemical pregnancy rate  Clinical pregnancy rate  Miscariiage rate  Endometrial thickness | No registration  No funding |
| Nazari 2019 IJ rep Biomed | Single center Iran | 60 women frozen | Inclusion criteria  age ≤ 38 years  BMI ≤ 30 kg/m2  history of cancelled FET cycle due to inadequate endometrial growth (≤ 7 mm) despite standard treatments  Exclusion criteria  uterine abnormalities, hormonal disorders, and hematological disorder | Protocol: two-step centrifuge process  Volume: 17.5 ml venous blood was drawn in the syringe that contained 2.5 ml of Acid Citrate Anticoagulant solution (ACD-A) (Arya Mabna Tashkhis, Iran) on cycle day 9 or 10.  Centrifugation: centrifuged immediately at 1200 rpm for 12 min to separate the red blood cells. The plasma was centrifuged again at 3300 rpm for 7 min | Control group (30 women)  Intrauterine infusion of sham_catheter was performed on day 11-12 due to the thin endometrium (thickness < 7 mm).  Intervention group (30 women)  Intrauterine infusion of 0.5 ml PRP or was performed on day 11-12 under ultrasonographic guidancedue to the thin endometrium (thickness less than 7 mm), and it was repeated after 48 hr if required | Chemical pregnancy rate  Clinical pregnancy rate  Endometrial thickness | IRCT2016072229027N1  Funded by the Research Department of the School of Medicine, Shahid Beheshti University of Medical Sciences (Grant no. 6776) |
| Nazari 2019 Hum fert | Single center Iran | 97 frozen | Inclusion criteria  age ≤ 40 years  BMI ≤ 30 kg/m2  failed to conceive after 3 or more embryo transfers with high-quality embryos and candidates for frozen thawed embryo transfer  Exclusion criteria  uterine abnormalities (congenital or acquired), hormonal disorders, immunological and haematological disorders, azoospermia, testicular sperm extraction or aspiration, anatomical disorders of the male genital tract, varicocele and chromosomal abnormalities in the couples | Protocol: Two-step process  Volume: 8.5 mL of venous blood added to 1.5 ml of acid citrate as the anticoagulant solution (ACD-A) (Arya Mabna Tashkhis, Iran)  Centrifugation: centrifuged immediately at 1200 rpm for 10 min to separate the red blood cells. The solution was centrifuged again at 3300 rpm for 5 min.  Platelet concentration: 4–5 times higher than circulating blood  There was no inactivation step during PRP production. | Control group (48 women)  No intervention  Interv4ntion group (49 women)  0.5 ml of PRP was infused into the uterine cavity with ET catheter under ultrasound guidance | Chemical pregnancy rate  Clinical pregnancy rate  Overlap with above study I duration registration number and fund | IRCT2016072229027N1Funded by the Research Department of the School of Medicine, Shahid Beheshti University of Medical Sciences (Grant no. 6776) |
| Nazari 2022 | Single center Iran | 393 frozen | Inclusion criteria  women had a history of failure to achieve pregnancy after three or more embryo transfers with high-quality embryos. Age between 18 and 38, body mass index (BMI) ≤30 kg/m2 , and serum FSH level ≤10 mIU/ml on day 2 or 3 of the menstrual cycle  Exclusion criteria  immunological abnormalities, inflammatory conditions, hormonal or anatomical disorders, polycystic ovary syndrome (PCOS), ovarian hyperstimulation syndrome (OHSS), endometriosis, presence of space-occupying lesions, history of miscarriage or ectopic pregnancy, myomas, polyps, adhesions, previous pelvic surgeries, failed fertilization, and less than two embryos available for transfer, severe male factor of their spouses and chromosomal abnormalities | Protocol: Two-step process  Volume: 8.5 mL of venous blood added to 1.5 ml of acid citrate as the anticoagulant solution (ACD-A) (Arya Mabna Tashkhis, Iran)  Centrifugation: centrifuged immediately at 1200 rpm for 12 min to separate the red blood cells. The solution was centrifuged again at 3300 rpm for 5 min.  Platelet concentration: 4–5 times higher than circulating blood | Control group (197 women)  the standard treatment No intervention  Intervention group (196 women)  Forty-eight hours before blastocyst transfer, received 0.5 ml of PRP through intrauterine infusion | Chemical pregnancy rate  Clinical pregnancy rate  Live birth rate  Multiple pregnancy rate | IRCT2016072229027N1  No fundings |
| Obidniak 2017 | ?? centers Russia | 90 | Inclusion criteria  women aged 28 - 39 years were involved Matching criteria: RIF, normal karyotype, absence of uterine factors of infertility, absence of chromosomal abnormalities in previous pregnancy  Exclusion criteria | Not described | Control group (45 women)  no therapy  Intervention group (45 women)  single IP with 2.0 ml of autologous PRP | Implantation rate  Clinical pregnancy rate  Miscarriage rate  Endometrial thickness  Adverse effects | No registration  Unknown funding |
| Rageh 2019 | Single center Bahrain | 150 fresh | Inclusion criteria  150 infertile women with history of RIF , age < 40 yrs., BMI < 30 kg/m2.  Exclusion criteria  The exclusion criteria were hematological and immunological disorders, hormonal disorders, chromosomal and genetic abnormalities and uterine abnormalities (acquired or congenital) | Protocol: Two-step process  Volume: 17.5 mL of venous blood added to 2.5 ml of acid citrate as the anticoagulant solution  Centrifugation: centrifuged immediately at 1200 rpm for 12 min to separate red blood cells, then plasma was centrifuged again at 3300 rpm for 7 min  Platelet concentration: 4-5 times more than peripheral blood | Control group (75 women)  No PRP  Intervention group (75 women)  intrauterine infusion of 0.5- 1 ml of PRP was infused into the uterine cavity with embryo transfer catheter 48 hours before ET | Chemical pregnancy rate | NCT04058783  No funds |
| Safdaria 2022 | Single center Iran | 120 frozen | Inclusion criteria  Age 20-40 years old, BMI < 30 kg/m2 women who failed to conceive after three or more ET with high quality embryos and had at least one frozen good-quality blastocyst-stage embryo, and were candidates for FET  Exclusion criteria  chromosomal and genetic disorders, hematological and immunological disorders, hormonal disorders, uterine abnormality (congenital or acquired), severe endometriosis, and patients with cancellation history of the previous ET due to a thin endometrium (≤7 mm) in hormone replacement therapy cycles | Protocol:  Volume: 8.5 mL of cubital vein blood added to 1.5 ml of acid citrate as the anticoagulant solution (Rooyagen, Iran)  Centrifugation: immediately centrifuged at 1600 rpm for 10 minutes to separate red blood cells. Then, plasma was re-centrifuged at 3500 rpm for 6 minutes at room temperature (18C) to obtain 1.5 mL lympho PRP  Platelet concentration: 4-5 times higher than the basal blood sample and 2000 lymphocyte/μL | Control group (60 women)  ET without the intrauterine infusion of PRP  Intervention group (60 women)  0.5 mL of PRP was gently infused into the uterine cavity with an intrauterine insemination (IUI) catheter under ultrasound guidance in sterile conditions | Implantation rate  Chemical pregnancy rate  Clinical pregnancy rate  Ongoing pregnancy rate  Live birth rate  Miscarriage rate  Multiple pregnancy rate | No registration  Funded by Tehran University of Medical Sciences |
| Zamaniyan 2020 | Single center Iran | 120 frozen | Inclusion criteria  120 patients, who failed to be pregnant after three or more embryo transfer of embryos with good quality and who underwent frozen-thawed embryo transfer  age 20–40 years, BMI < 30 kg/m2 , and normal hysterosalpingography  Exclusion criteria  hematologic disorders (blood cancer, thrombocytopenia), immunologic disorders (anti-phospholipid syndrome, thrombophilia), hormonal disorders (diabetes, thyroid, hyperprolactinemia), chromosomal and genetic anomalies (hereditary or congenital), and renal failure | Protocol:  Volume: 17.5 mL of venous blood added to 2.5 ml of acid citrate as the anticoagulant solution (Arya MabnaTashkhis, Iran)  Centrifugation: centrifuged immediately at 1200 rpm for 12 min to detach RBCs then plasma was recentrifuged at 3300 rpm for 7 min  Platelet concentration: 4–7 times more than peripheral blood | Control group  Intervention group  0.5 ml of platelet-rich plasma was infused into the uterine cavity with intra uterine insemination catheter | Chemical pregnancy rate  Clinical pregnancy rate  Implantation rate | IRCT201608150 29374N2  No funding |
| Zaragar 2021 | ?? centers Iran | 80 (14 fresh ,66 frozen) | Inclusion criteria  Infertile women with at least two IVF failures and age < 41 years old  Exclusion criteria  women with chromosomal, genetic, and uterine abnormalities, hematological or immunological disorders, and hormonal disorders and the embryos that arise from such maternal and paternal abnormalities | Protocol: Fertilize Lympho-PRP kit (PRP centrifuge kit; Prodizen, Korea)  Volume: 8.5 mL of venous blood added to 1.5 ml of acid citrate as the anticoagulant solution (ACD; Terumo; at a ratio of 10:1.5)  Centrifugation: Centrifuged immediately at 12,000X g for 10 min. The blood was divided into 3 layers: bottom RBCS, supernatant cellular plasma and intermediate buffy coat layer. The plasma layer was collected to another tube and re-centrifuged at 12,000X g for 10 min. Platelet pellets white sediment that was formed at the bottom  of the tube that was dissolved in 1.5 mL plasma [the 1.5-line indicated  on the tube for infertility cases] was used as PRP containing platelet growth factor. | Control group (40 women)  No PRP  Intervention group (40 women)  PRP (1.5 mL) was injected into all patients’ endometrium whether they had an appropriate endometrial thickness (7-10 mm) or not (< 7 mm). Intrauterine infusion of PRP was performed 48 h before embryo transfer in those who had an appropriate endometrial thickness; however, PRP (1.5 mL) was re-injected for two patients who did not have an appropriate endometrial size 48 h after the first injection [11]. After sonographic reassessment, the embryo transfer process was done by Frydman catheter | Successful pregnancy rate  Miscarriage rate  Live birth rate | IRCT20180102038182N1  funded by Ahvaz Jundishapur University of Medical Sciences |
| Apolikhina 2021 | Single centers (author correspondence)  Russia | 68 frozen | Inclusion criteria  age 18–39, normal karyotype of the patient and her partner, regular ovulatory menstrual cycle, endometrium thickness no more than 7 mm during ''implantation window'  Exclusion criteria  : marked pathospermia in a partner, use of donor gametes, preterm ovarian insufficiency, internal genitalia anomalies, contraindications for physical factors treatment, systemic blood diseases and coagulopathy, Hb level less than 100 g/l, platelet level less than 100х109/l, antiplatelet and anticoagulant therapy | Not described | control group (n=30) treated using only physiotherapeutic methods  Intervention group (n=38) were prepared for embryo transfer in two steps: physical treatment course (the first cycle), autologous 35–40 ml of autologous PRP injections into endometrium at a depth of 2–3 mm on the day 6–8 under intravenous anesthesia and hysteroscopic control. 6–8 targeted PRP injections were made into the most defective areas of the uterine mucosa | s endometrium thickness and structure as well as uterine hemodynamics after the therapy | NCT05455151 (author correspondence)  No funds |
| Eftekhar 2018 | Single center Iran | 83 frozen | Inclusion criteria  women candidated for FET because of poor endometrial response (endometrium thickness < 7 mm) to standard hormone replacement therapy (HRT) in the 13th day of the cycle in FET cycles with age between 18 and 42 years  Exclusion criteria  women with haematological, immunological or hormonal disorders chromosomal and genetic abnormalities and congenital or acquired uterine abnormalities | Protocol:  Volume: 8.5 mL of venous blood added to 1.5 ml of acid citrate as the anticoagulant solution (ACD-A) (Arya Mabna Tashkhis, Iran)  Centrifugation: centrifuged immediately at 1600 g for 10 min. The blood was divided into 3 layers bottom RBCS, supernatant cellular plasma and intermediate buffy coat layer. The plasma layer and buffy coat were collected to another tube and centrifuged again at 3500 g for 5 min to obtain 1.5 ml PRP  Platelet concentration: 4-5 times more concentrated and 2000 lymphocyte | Control group (n=43)  treated with received HRT  Intervention group (n=40)  treated with HRT and intrauterine infusion of 0.5 -1 cc PRP on the 13th day of HRT cycle. | Endometrial thickness  Chemical pregnancy rate  Clinical pregnancy rate  Ongoing pregnancy rate | IRCT2016090328950N2  No funds |

Table S2 characteristics of Non RCTs

| [Study] | [Settings] | [Sample size] | [Participants] | PRP preparation | [Interventions] | [Outcomes] | [Notes] |
| --- | --- | --- | --- | --- | --- | --- | --- |
| Noushin 2021 | Single center UK | 318 frozen | Inclusion criteria  Women aged <40 years with a history of RIF undergoing  frozen ET  Exclusion criteria  BMI ≥ kg/m2, congenital and untreated acquired uterine  abnormalities, untreated hydrosalpinges, poor ovarian  responder as per the Bologna criteria, thrombophilia, or uncontrolled  endocrine or hematologic dysfunction; those undergoing  preimplantation genetic testing cycles; and those  who had thin endometrium (<8 mm) in the index FET cycle.  Severe male factor infertility, difficult ET, only poor-quality  embryos available, and couple with genetic and chromosomal  abnormalities | Protocol: Standardized with FDA approved commercially available kit (Regen PRP kit).  Volume: 60 mL of venous blood was divided into 10-mL aliquots each into four acid–citrate–dextrose solution gel vacutainer tubes to prevent platelet activation.  Centrifugation: centrifuge immediately after collection at 1,200 rpm for 10 min at 20 -22 °C to separate RBCs from plasma.  Plasma was recentrifuged at 3,000 rpm for 10 min to obtain platelet concentrate. The supernatant plasma was discarded, and the bottom platelet pellet was then made into aliquots inside two cryowells; this was then well capped and tightened. For platelet activation, a chemical-free activation protocol using liquid nitrogen (the platelet concentrate was subjected to 2-3 cycles of freezing and thawing | Subendometrial (SE-PRP group,  N = 55) were administered subendometrial injection of autologous  activated PRP in the luteal phase of the previous cycle  of ET between cycle days 21 and 24.  Intrauterine PRP (IU-PRP group, N = 109) were administered  autologous intrauterine activated PRP during the index FET  preparation cycle.  (Control group, N =  154)Participants who did not choose for PRP  treatment. | Ongoing pregnancy rate or live birth rate (OPR/LBR) per transfer cycle, clinical pregnancy rate (CPR) per  transfer cycle, and miscarriage rate. | No registration  Funded by a private tertiary level clinic and a couple who paid the cost of the treatment. |
| Chang 2019 | Single ceter China  Pro Cohort | 64 thin edomet frozen | Inclusion criteria  Patients with thin endometrium who were  going to receive FET  (1) age younger than 40 years old, and basal serumFSH<10IU/L;  (2) with cancellation history of embryo transfer due to thin  endometrium (<7mm) in hormone replacement therapy  (HRT) cycles;  (3) no obvious intrauterine adhesion in the diagnostic hysteroscopy;  (4) no submucosal uterine myoma or endometrial polyps;  (5) no history of hematological disorders (e. g., leukopenia,  thrombasthenia et al);  (6) have at least 2 frozen good-quality blastocyst-stage embryos.  Exclusion criteria  history of pelvic cancer,  severe endometriosis, and adenomyosis. | Volume: 15 mL of venous blood mixed with 5 mL anticoagulant solution (ACD-A)  Centrifugation: centrifuged immediately at 300 X g for 10 minutes at 18°C. 3 layers were formed bottom RBCS, supernatant cellular plasma and intermediate buffy coat layer. The plasma layer and buffy coat were collected to another tube then centrifuged at 700 Xg for another 15 minutes at 18°C. After discarding about 3/4 of the supernatant, 0.5 to 1mL of PRP was pipetted from the bottom of tube. The PRP was activated by adding a mixture of thrombin powder (25IU/mL; Sigma-Aldrich, St. Louis, MO) and calcium chloride (20 mmol/mL; Kalmia, Korea United) at 18°C. | Control group ( n=30)  No intervention  PRP group (n=34 patiets 42 cycles)  0.5 – 1 ml intrauterine infusion on the  10th day and the day when progesterone was given in HRT cycle with Tomcat  catheter | endometrial thickness. The second  endpoints are implantation rate and clinical pregnancy rate | Funded by the National Natural Science  Foundation of  ChinaCREATE 2014, the Doctoral Fund of the Ministry of Education of  China (No.20090171110059), Natural Science Foundation of Guangdong  Province (No.2014A030310096, 2015A030313013) and Public Welfare Research  and Capacity Building Fund of Guangdong |
| Dzhincharadze 2021 | ?? Russia | 54 fresh | Inclusion criteria  age 20–42 years; regular menstrual cycle (25–34 days); body mass index: 18–30 kg/m2; infertility due to tubal and/or male factor and/or external genital endometriosis; idiopathic infertility; the history of embryo transfer cancellation due to "thin" endometrium; normal uterine cavity confirmed by hysteroscopy; at least 3 vitrified blastocysts: 1) excellent quality; 2) good quality and/or average quality  Exclusion criteria  history of cancer, severe external genital endometriosis, intrauterine pathology or congenital malformations of the uterus before surgical treatment | On cycle day 8, 400.0 ml of autologous blood was collected in the first container of the integrated "Blood bag" system (JMS Singapure) with 63 ml of the anticoagulant CPDA. Next, the blood container was centrifuged in a Becman refrigerated centrifuge for 8 minutes in the 1971g mode at a temperature of + 22°C. After centrifugation with a plasma extractor, the plasma and platelets (upper and middle layer) were moved to the second container. The container with autoerythrocytes was detached, and they were reinfused to the patient. The second container with plasma and platelets was re-centrifuged for 10 minutes with a centrifugal acceleration of 5130g at a temperature of + 22°C. After that, using a plasma extractor, the upper layer – native plasma-was removed to the third container, and the lower layer, concentrated platelets suspended in plasma, was moved to a special container for platelets storage (JMS, Singapure). The container with PTP was transferred to the climatic chamber for mixing platelets of LmB Technologie GmbH (Germany), where it can be stored for 5 days at a temperature of +22–24°C. | control group (17 patients ) with "thin" endometrium who received only cyclic hormone therapy  Intervention group (37 women ) received intrauterine PRP on the 8–9, 10–11, and 12–13 days of the menstrual cycle with cyclic hormone therapy | Endometrial thickness  Clinical pregnancy rate | No registration  No funds |
| Tehraninejad 2020 | Single center Iran | 85 frozen | Inclusion criteria  85 women with RIF who were admitted for frozen ET  Exclusion criteria  age ≥35 years, endometrial thickness 10 mIU/mL, severe male factor such as azoospermia, intrauterine disorders, thrombophilia, thyroid dysfunction, positive antiphospholipid antibodies or chromosomal abnormality in a couple | Volume: 10 mL blood were drawn in a 20-mL syringe, containing 1.5 mL of acid-citrate-dextrose solution (Arya Mabna Tashkhis)  Centrifugation: Blood was transferred into 50-mL falcon tubes and centrifuged for  10 min at 1200 rpm for sedimentation of RBCs. The supernatant solution was extracted and then centrifuged at 3300 rpm for 5 min.  Platelet concentration: 4-5 times higher than the circulating blood | Control group  Intervention group  1 mL of PRP was injected into the uterine cavity under ultrasound guidance. Two days later, ET was carried out using an ET catheter under ultrasound guidance | Chemical pregnancy rate  Clinical pregnancy rate  Ongoing pregnancy rate | No registration  No funding |
| Kim 2019 | Single center South Korea | 24 frozen | Inclusion criteria  (a) age 20–45 years  (b) endometrial thickness <7mm  (c) ≥ 2 failed IVF cycles,  (d) ≥ 2 cycles of previous therapy for increasing  the EMT, such as, hysteroscopic adhesiolysis following hormone  replacement therapy, high dose estradiol valerate, transvaginal  sildenafil administration, or pentoxifyilline combination with  vitamin E, (f) frozen embryo available for ET, and (g) informed  consent form signed. Exclusion criteria  (a) hematologic disorders, hemoglobin level of <9.0 g/dL or  platelet count of <100,000/μL, (b) auto-immune disease, (c)  chromosomal abnormality in the patient or spouse, (d) peripheral  NK cell proportion of ≥12%, (e) body mass index (BMI) of  ≥30 kg/m2, and (f) uncontrolled endocrine or other medical  conditions, such as prolactinemia or thyroid diseases. | Volume: 18 mL of venous blood was drawn using 30mL syringes coated with 2 cc of acid citrate A, anticoagulant solution (ACD-A; Arya Mabna Tashkhis, Iran).  Centrifugation: In aseptic PRP centrifuge kit (PROSYS PRP; Prodizen, Korea),the blood was centrifuged at 1017 G for 3 min. The buffy coat and plasma just above the buffy coat were collected, and 0.7–1.0 mL of PRP was produced and infused.  Platelet concentration: 717 - 1565 × 10^3^/μL, and the WBC concentration 24,000 - 37,000/μL | Intervention group intrauterine infusion of  autologous PRP 2 or 3 times from menstrual cycle day 10 of their frozen-thawed embryo  transfer (FET) cycle, and ET was performed 3 days after the final autologous PRP infusion  Control group same patients in previous cycle | ongoing pregnancy rate and LBR.  implantation rate, clinical pregnancy rate, and EMT  increment compared with those on the previous cycle | KCT0003375 registration  Supported by a grant of the Research Driven  Hospital R&D project, funded by the CHA Bundang Medical  Center (grant number :BDCHA R&D 2015-47) |
| Kusumi 2020 | 7 fertility clinics Japan | 39 included in safety and 36 for efficacy Frozen | Inclusion criteria  age of 20-50 years, oocyte retrieval performed at age 42 years or younger, and endometrial thickness of ≤7 mm  Exclusion criteria  hepatic disorders, hemoglobin level of | Volume: 20 mL Peripheral blood was drawn from the forearm in 2 tubes (10 mL for each tube) using vacuum blood collection tubes (Acti-PRP tube, Aeon International Inc, Taipei, Taiwan).  Centrifugation: centrifuged at 2000 *g* for 6 minutes | transdermal or oral estrogen tablets were used with a stable or step-up regime from day 2 -3 twice . Endometrial thicknesses were measured on D1,3,10.12.14 of the first and second HRT cycles. The D14 endometrial thicknesses of the second HRT cycle were compared with D14 of the first HRT cycle. Hormonal measurements and PRP intrauterine infusion were both performed on the 10th and 12th day of the second HRT cycle. Subsequently, vaginal progesterone suppository therapy was started. FET was performed on the 19th day or the appropriate date of the second HRT cycle | Endometrial thickness  Adverse events  Pregnancy rate  Implantation rate | No registration  funded by Aeon International Inc |
| Dogra 2022 | Single center India | 20 women 26 cycles  14 fresh and 12 frozen | Inclusion criteria  Infertile women of age less than 38 years who have EMT persistently less than 7mm on baseline endometrial evaluation before IVF despite standard HRT and hysteroscopically normal endometrial cavity and women with a history of cycle cancellation during FET cycles due to persistent thin endometrium  Exclusion criteria  women with distorted endometrial cavity because of recurrent adhesions, submucous fibroids, endometrial polyps; severe endometriosis; adenomyosis and uterine anomalies | Protocol: Two-step centrifuge process  Volume: 15 mL of venous blood were drawn in a vial containing anticoagulant solution ACD-A (Anticoagulant Citrate Dextrose Solution, Solution A)  Centrifugation: centrifuged immediately at 175g for 12 minutes to separate the red blood cells. The plasma and buffy coat were collected in another empty tube and centrifuged again at 1300g for 7 minutes. The resulting pellet of platelets was mixed with 1 ml of supernatant and 0.5-1ml of PRP was instilled into the uterine cavity  Platelet concentration: above 1,000,000/μL | HRT in the form of estradiol valerate 6mg/day orally starting from D2 . On day 8. If EMT was < 7mm, PRP was prepared and infused into the uterine cavity using an intrauterine (IUI) catheter. EMT and pattern were reassessed every 48 hours, and PRP infusion was repeated if required. When EMT reached more than 7mm, Injection progesterone 100 mg intramuscular daily was started, and ET was performed on day 3 or 5 accordingly. The patients were recruited in the present study for fresh IVF only when EMT reached 7mm. IVF was done according to the flexible GnRH antagonist protocol. During the stimulated cycle, if adequate EMT was obtained (>7mm) during ovarian stimulation, the cycle was completed as per protocol, and no PRP was infused. If EMT was less than 7mm on day 8, intrauterine PRP was administered. EMT was reassessed after 48 hours, and PRP infusion was repeated if required. The maximum number of PRP infusions was limited to three. The cycle was completed with ovulatory trigger (hCG or dual) when 2-3 follicles achieved 18mm diameter followed by oocyte retrieval after 34-36 hours. Embryo transfer was carried out on day 3 or 5 depending on embryo quality if EMT was more than 7mm on the day of trigger. | Endometrial thickness  implantation rate (IR), clinical pregnancy rate (CPR) and LBR in fresh and FET cycles and aetiology wise | registered prospectively (CTRI/2018/12/016799)  No funds |
| Wang 2018 | Single center China | 20 frozen | Inclusion criteria  Twenty patients between 27 and 43 years of age undergoing frozen ET All had < 7mm max endometrial thickness despite receiving a standard dose of estradiol valerate (up to 16 mg/day) or suboptimal endometrial vascularity <5 vascular signals reaching the central endometrial zone and cancelled fresh ET. Women with RIF with poor endometrium and women with >2 cancelled cycles due to poor endometrium  Exclusion criteria  any other known cause of implantation failure, such as poor embryo quality, congenital uterine anomalies, or Asherman syndrom | Volume: PRP was collected from four donors using citrate dextrose solution as anticoagulant  Centrifugation: centrifuged at 200 × g for 15 minutes followed by hard spin of 300 × g for 10 minutes at room temperature to discard the supernatant. Subsequently,  PRP was mixed with an equal volume of thrombin solution (5 U/mL in 40mM CaCl2 buffer, Baxter, Munich, Germany) for activation about 1 hour. Following activation,  PRP was centrifuged for 10 minutes at 2000 × g to remove cellular debris and collected through a 0.2 μm filter. Activated PRP were stored at −80°C for use  Platelet concentration: 1012/L | estradiol valerate started from the first day of their menses in a dose of 6 to 8 mg/day, and gradually increased up to 12 mg/day as needed, and failed to achieve endometrial thickness≥7 mm despite using optimal dose of estradiol valerate for 15 to 16 days. Professional doctors infused a volume of 0.5 to 0.8 mL of the PRP to patients’ intrauterine using an intra‐uterine insemination (IUI) canula under ultrasound guidance following all aseptic precautions | Endometrial thickness | Natural Science Foundation of Shandong Province, Grant/Award Numbers: ZR2016hl08, ZR2017PH047 |
| Zadehmodarres 2017 | Single center Iran | 10 frozen | Inclusion criteria  history of cancelled cycles due to inadequate endometrial growth (less than 7 mm) in the past FET cycles despite standard treatments  Exclusion criteria  Unclear | Protocol: Two-step centrifuge process  Volume: 17.5 ml of peripheral venous blood was drawn in the syringe that contained 2.5 ml of Acid Citrate Anticoagulant solution (ACD-A) (Arya Mabna Tashkhis, Iran) on cycle Day 9 or 10  Centrifugation: centrifuged immediately at 1200 rpm for 12 min to separate the red blood cells. The plasma was centrifuged again at 3300 rpm for 7 min to obtain the PRP | estradiol valerate 6 mg/d was started on the 2nd or 3rd day of the mensural cycle and it was increased to 8 mg/d on day 9-10 because of inadequate endometrial growth (< 7 mm). PRP was performed on day 11-12 in all the patients due to thin endometrium and it was repeated on day 13-14. During the cycle, whenever the endometrial thickness was more than 7 mm, suppository progesterone 400 mg twice-aday was started and embryo transfer (ET) was carried out per embryonic stage. Estradiol valerate and progesterone supplementation were continued for 2 weeks after ET and if the serum βHCG was positive, hormone supplementations were continued until 12 weeks of gestation. | Endometrial thickness  CPR  Chemical pregnancy rate | No registration funded by the Research Department of the School of Medicine Shahid Beheshti University of Medical Sciences (Grant no 6776) |
| Madhavan 2018 | Single center India | 98 frozen  42 PRP  56 control | Inclusion criteria  Patients aged 25–40 years undergoing FET who had at least one previous FET failure with two good cleavage stage embryos or blastocysts, derived from autologous oocytes in an endometrium 7 mm or more in thickness  Exclusion criteria  Known uterine abnormalities, previous history of endometrial disease such as atypia or Asherman’s disease, known genetic diseases, cancer, comorbid conditions which might affect IVF success, nonconsented patients | Volume: 15 ml of peripheral venous blood was added to 5 ml of Acid Citrate A Anticoagulant solution  Centrifugation: centrifuged immediately at 200 g for 10 min to separate the red blood cells. The plasma and buffy coat obtained were centrifuged again at 500 g for 8 min to obtain 0.3–0.4 mL of PRP. | All patients underwent the same HRT regimen for endometrial preparation. Estradiol valerate (E2V) was started in a dose of 4 mg orally from the 2nd or 3rd day of the menstrual cycle and was escalated to 6 mg after 4 days. The maximal dose of E2V given was 12 mg  Control group 56 women did not receive any PRP  study group had 42 patients who received intrauterine infusion of PRP on day 8/9 of the hormone replacement therapy (HRT) cycle | CPR | No registration No funds |
| Xu 2022 | Single center China | 288 RIF frozen  138 PRP  150 control | Inclusion criteria  Patients aged 23 to 40 years who had three or more consecutive failed embryo implantations with good-quality embryos (at least 6 cleavage-stage embryos or three blastocysts)  Exclusion criteria  an abnormal karyotype from either partner, evidence of uterine defects, ultrasonographic evidence of hydrosalpinx, infections, endocrine problems, coagulation defects or autoimmune defects | Volume: 20 mL of peripheral blood  Centrifugation: centrifuged at 500 × g for 10min at 18◦C. After centrifugation, 3 layers appear (serum, platelets, and RBCs). The serum and platelets were transferred to another tube and recentrifuged at 500×g for 10min at 18◦C. The supernatant was discarded, the remaining 1mL PRP was infused.  Platelet concentration: 1513.45 ± 322.18 × 10^9^/L | Control group 150 women  Endometrial preparation for frozen-thawed cycle included hormone replacement treatment (HRT) protocol (estrogen progesterone cycle, EP) and natural cycle (NC). One or two good quality embryos were transferred  PRP group 138 women beside endometrial preparation  1 mL PRP was infused into the uterine cavity using artificial insemination catheter 2 days | LBR  CPR  Chemical pregnancy rate  Miscarriage rate  Implantation rate | No registration  Funded by Qingdao Key Health Discipline Development Fund |
| Coksuer 2019 | Single center Turkey | 273 women  302 cycles  70 analyzed | Inclusion criteria  history of RIF ( ≥ consecutive IVF), normal hysteroscopy results, normal karyotype of both couple, a regular menstrual cycle of 21–35 days, evidence of ovulation ,D2 FSH <10 IU/L, normal tubal patency, normal semen parameters, Age 21-39 years, BMI 18-28 kg/m2  having three blastocyst FET and could not be able to achieve optimal endometrium < 7 mm despite appropriate estradiol valerate treatment  Exclusion criteria  any systemic, immunologic, endocrine disease and thrombophilia, donor eggs, previously taken PRP as an infertility therapy, patients with poor embryo quality or had RIF history due to Asherman’s syndrome or congenital uterine anomalies | Protocol: The standard protocol of American Association of Blood Banks  Volume:8mL of venous blood were clooected by peripheral venipuncture to tubes (Regen Lab, Mont-sur-Lausanne, Switzerland).  Centrifugation: immediate centrifugation at room temperatureat a rate of 1500 g for 5 min. After centrifugation, the blood was fractionated; RBCs were trapped under the gel while lower density components stratified above the surface of the separator  gel. Supernatant platelet-poor plasma fraction was discarded before recapping the vial for gentle tube inversion/resuspension. Remaining 1mL PRP was infused with IUI cannula under ultrasound guidance.  Platelet concentration: 992.45 ± 212.85 X 10^3^ cell/mL | Control 36 women Hormone replacement therapy was performed for endometrial preparation by estradiol valerate from day 1 of the menstrual cycle in a dose of 6 mg/day and it was increased to 12 mg/day if endometrial thickness was <7 mm  PRP group 34 women  g 1 mL PRP was infused with IUI cannula under ultrasound guidance in the same FET cycle, 48 h before FET | Endomterial thickness  CPR  Chemical pregnancy rate  LBR  Miscarriage rate  Ectopic pregnancy | No registration No funds |
| Enatsu 2021 | Single center japan | 54 cycles frozen | Inclusion criteria  RIF, defined as having a history of at least two consecutive cycles of implantation failure and 25–45 years of age during ET. To exclude the influence of embryo quality, we only analyzed ETs with high-quality blastocyst  Exclusion criteria  hepatic disorder, hemoglobin level < 11g/dl, platelet count < 150,000/mm3 | Volume: 20 ml of peripheral blood from the forearm was collected using 2 vacuum  blood collection tubes (Acti-PRP tube, Aeon International Inc.),  Centrifugation: at 2000 *g* for 6 min results in bottom WBCs and RBCs layer, supernatant plasma layer and middle buffy coat layer. One ml (0.5 ml per tube) of PRP was obtained from the bottom of the supernatant layer | Administration of transdermal and oral estradiol was started on cycle day d intrauterine PRP infusion was performed on CD 10 (first time) and CDs 12–14 (second time)  Compared to 187 women in previous cycles with high-quality blastocysts at the same CD | CPR  Chemical pregnancy rate  Miscarriage rate  Ongoing pregnancy rate | Registered (clinical study No. PB5190013  No funds |
| Russell 2022 | Single center Canada | 85 patients 133 treatment cycles and 211 PRP infusions  23 thin endometrium  48 RIF  14 RIF & thin endometrium | Inclusion criteria  Patients aged 24–52, diagnosed with recurrent implantation failure and/or persistent thin endometrium who had PGT-A-tested euploid embryos and received one or more intrauterine PRP infusions  Exclusion criteria  an inactive endometrium, multiple embryos transferred, genetic abnormalities, hematologic disorders, or an autoimmune disease. Diploid-aneuploid mosaic embryo transfer | Protocol: Two step centrifugation method  Volume: 21 cc of blood was drawn into ACD-A tubes  Centrifugation: centrifuged at 900 relative centrifugal force (rcf) and 14 °C for 10 min. The upper layer containing the plasma and platelets was transferred to a new tube; the WBCs and RBCs layers were discarded. The plasma was recentrifuged  at 1500 rcf and 14 °C for 15 min to pellet the platelets. The platelet-poor plasma was discarded, leaving 0.5–0.75 cc to resuspend the platelets. The PRP was stored at 4 °C for 2 h or less until infusion. | Treatment with autologous PRP was performed in estrogen primed FET cycles. Administration of the PRP was performed between cycle days 10–15 and patients with an EMT remaining < 7 mm received infusions until the lining reached over 7 mm in thickness. The PRP was aspirated into a Tomcat catheter and infused into the uterus under ultrasound guidance | CPR  Chemical pregnancy rate  Miscarriage rate  LBR | No registration  funded by the CReATe Fertility Centre through reinvestment of clinical earnings |
